# PSA-FL-CDM: A Novel Federated Learning-Based Consensus Model for Post-Stroke Assessment

**DOI:** 10.3390/s24165095

**Published:** 2024-08-06

**Authors:** Najmeh Razfar, Rasha Kashef, Farah Mohammadi

**Affiliations:** Electrical, Computer, and Biomedical Engineering, Toronto Metropolitan University, Toronto, ON M5B 2K3, Canada; nrazfar@torontomu.ca (N.R.); fmohamma@torontomu.ca (F.M.)

**Keywords:** consensus clustering, camera-base dataset, federated learning, PSA_MNMF model, stroke assessment, wearable datasets

## Abstract

The rapid development of Internet of Things (IoT) technologies and the potential benefits of employing the vast datasets generated by IoT devices, including wearable sensors and camera systems, has ushered in a new era of opportunities for enhancing smart rehabilitation in various healthcare systems. Maintaining patient privacy is paramount in healthcare while providing smart insights and recommendations. This study proposed the adoption of federated learning to develop a scalable AI model for post-stroke assessment while protecting patients’ privacy. This research compares the centralized (PSA-MNMF) model performance with the proposed scalable federated PSA-FL-CDM model for sensor- and camera-based datasets. The computational time indicates that the federated PSA-FL-CDM model significantly reduces the execution time and attains comparable performance while preserving the patient’s privacy. *Impact Statement*—This research introduces groundbreaking contributions to stroke assessment by successfully implementing federated learning for the first time in this domain and applying consensus models in each node. It enables collaborative model training among multiple nodes or clients while ensuring the privacy of raw data. The study explores eight different clustering methods independently on each node, revolutionizing data organization based on similarities in stroke assessment. Additionally, the research applies the centralized PSA-MNMF consensus clustering technique to each client, resulting in more accurate and robust clustering solutions. By utilizing the FedAvg federated learning algorithm strategy, locally trained models are combined to create a global model that captures the collective knowledge of all participants. Comparative performance measurements and computational time analyses are conducted, facilitating a fair evaluation between centralized and federated learning models in stroke assessment. Moreover, the research extends beyond a single type of database by conducting experiments on two distinct datasets, wearable and camera-based, broadening the understanding of the proposed methods across different data modalities. These contributions develop stroke assessment methodologies, enabling efficient collaboration and accurate consensus clustering models and maintaining data privacy.

## 1. Introduction

Internet of Things (IoT) advancement technologies have revolutionized conventional healthcare and rehabilitation systems by facilitating remote consultations through smart rehabilitation and health systems [1,2]. Wearable devices and camera-based systems play a vital role in smart rehabilitation by providing an interface for IoT-enabled technology, artificial intelligence, and machine-learning techniques [3,4]. Wireless sensors and portable motion capture systems are equipped with multiple sensors, such as inertial measurement units (IMUs), that continuously record patients’ movements, which provides useful information for assessing patients’ motions and ability to move [3]. A multitude of data is continuously generated from diverse devices like wearable sensors, camera systems, IoT devices, and smartphones. For many years, centralized data analysis has been practiced in various domains, such as scientific research, business intelligence, and health research. It was further popularized when big data technologies and cloud computing were introduced. However, centralized data analysis suffers from various drawbacks, such as privacy and security risks, especially when the patients’ datasets for health or rehabilitation are investigated. In addition, it requires an expensive infrastructure or investment in hardware and storage for a big dataset. In 2016, Google introduced federated learning (FL) to tackle these challenges, focusing on preserving privacy for sensitive healthcare data [5,6,7,8]. For healthcare and rehabilitation datasets, where the privacy and ethical considerations of sensitive data are of utmost importance, federated learning offers a solution. Unlike centralized learning environments that necessitate substantial memory and extensive computational time, federated learning addresses these concerns. With federated learning, each client’s raw data remain stored locally and are not shared or transferred. Instead, targeted updates designed explicitly for immediate aggregation are used to accomplish the desired learning objective. This approach ensures data privacy and security while enabling effective collaboration in learning. Federated learning has garnered significant interest in healthcare research due to its ability to address the critical issue of preserving patient privacy, given the limitations of expanding datasets. The incorporation of big data is essential for improving healthcare-related models. In healthcare, federated learning has been successfully applied to medical imaging modalities such as X-ray, MRI, and CT scans [9,10,11,12]. Notably, research has focused on brain tumor segmentation and the detection of COVID-19 using X-ray or CT-scan images [9,10,11,12]. These applications demonstrate the potential of federated learning to advance healthcare practices while maintaining patient privacy. In cardiac and stroke artificial intelligence (AI), federated learning has not played a role like it has in other research areas. To the best of the authors’ knowledge, no previous studies have automated stroke assessments using federated learning nor introduced consensus clustering methods to any healthcare domain. This research represents the first implementation of eight distinct unsupervised learning models within a federated learning framework. Moreover, it uniquely incorporates data collected through two different methods—camera-based and wearable sensors—to evaluate the effectiveness of federated learning results and deal with crowdsourced data due to the large, representative datasets. This research presents several novel contributions:

1. Federated learning has been successfully implemented, which allows multiple clients to collaborate and train a shared model without sharing raw data.
2. Multiple clustering methods have been applied independently to each client. This step aids in organizing and grouping data points based on similarities.
3. The centralized PSA-MNMF consensus clustering technique has been locally applied to each client, and then the outputs are combined to obtain a more robust solution.
4. FedAvg, a federated learning algorithm, has been utilized to aggregate and combine the locally trained models from different clients or nodes. This results in creating a global model that represents the collective knowledge of all participants while maintaining data privacy.
5. Notably, this research explores multiple input data sources across different data modalities.

This paper is organized as follows. Section 2 presents background and related work on federated learning. Section 3 offers the proposed federated learning model using PSAMNMF. In Section 4, a comprehensive discussion of the material and methods used in the study is presented. Section 5 presents a detailed description and analysis of the experimental results. Furthermore, Section 6 includes a thorough analysis and discussion of the results. In Section 7, the paper concludes by summarizing the key insights gained from the study and offers potential future directions.

## 2. Related Work and Background

Since 2016, federated learning has gained significant interest in healthcare research due to the vital need to protect patient privacy, limiting dataset expansion. Access to large-scale data is crucial for enhancing healthcare models. FL has found successful applications in analyzing medical images like those from X-rays, MRIs, and CT scans, particularly in tasks such as brain tumor segmentation and COVID-19 detection [9,13,14,15,16,17]. Working with medical image data, such as MRI and CT scans, for stroke prediction, brain tumor diagnosis, or COVID-19 detection holds promise for several reasons. Firstly, medical images generally adhere to standardized protocols worldwide. Secondly, obtaining ethical approval for using medical images is relatively more straightforward than other data types, like motion capture data. Lastly, deep learning techniques have already gained wide acceptance in the field of FL, further supporting their application in medical image analysis. The predominant approach observed in the research was utilizing the federated average algorithm (FedAvg). Machine learning techniques, primarily deep learning models, were mainly employed for supervised classification tasks. One study incorporated natural language processing and applied federated learning, focusing on horizontal FL [18]. The vertical federated learning models are also used in some studies [13,19]. The paper introduces a federated nonnegative matrix factorization (FedNMF) framework for training topic models collaboratively on locally stored data and addressing privacy concerns and proposes FedNMF + MI to maximize mutual information exchange between local text features and topic weight vectors, resulting in improved performance over standard federated learning methods like FedLDA, particularly in text classification tasks, as demonstrated through experimental results [20]. However, limited studies have explored federated transfer learning [17,21].

Federated learning also promises to revolutionize stroke care analysis and improvement, as strokes require prompt and specialized attention. With the rise of wearable devices, camera-based motion data, and the Internet of Things (IoT), real-time data collection from various sources, including clinical centers, hospitals, and rehabilitation centers, has become feasible. The work in [22,23] evaluated five deep-learning methods for predicting internal knee abduction impulses during walking using three-dimensional kinematic and kinetic data in wearable sensor technology and predictive healthcare analytics [22]. Transfer learning with inception time emerged as the top-performing model, achieving the lowest mean absolute percentage error (MAPE) of 8.28%, highlighting the superiority of time-series-based deep learning models over image-based approaches in predicting knee joint moments [22,23]. Another work presents a novel framework for accurately recognizing activities performed by stroke patients using wearable sensors. By combining deep learning models and leveraging a data fusion mechanism based on multiple sensors, the proposed approach effectively captures temporal patterns and dependencies, resulting in improved activity classification performance. Experimental results on benchmark datasets demonstrate the superiority of the proposed model over baseline and alternative methods, highlighting its potential for enhancing post-stroke rehabilitation systems [24]. Lastly, another study aimed to assess the predictive value of high-resolution data from wireless, wearable motion sensors in determining post-stroke ambulation function after inpatient rehabilitation. Supervised machine learning algorithms trained on inertial measurement unit (IMU) sensor data recorded during walking bouts at admission improved the prediction of discharge ambulation ability, showing potential for early prognostication of functional recovery in stroke patients and aiding in the design of personalized care strategies [25].

Utilizing the power of federated learning with this diverse data presents an unprecedented opportunity to advance stroke care. In [26], they used federated learning to develop a machine-learning model for predicting stroke risk using wearable sensor data from multiple sources. The federated learning approach enabled the model’s training on distributed and diverse wearable data sources without transferring the sensitive data to a centralized location. The results demonstrated that the federated learning approach achieved accuracy similar to that of a centralized approach while maintaining data privacy and security. Unfortunately, the research lacked documentation of computational time and various performance metrics, including accuracy, precision, recall, and F1-score. Only the study by [18] reported all these metrics except accuracy. To our knowledge, no study has focused on stroke assessments deploying federated learning, and no consensus clustering has been implemented as an ML model in local training. Overall, federated learning holds great promise for improving stroke assessment by enabling the training of machine learning models on distributed and diverse data sources while preserving data privacy and security. By leveraging the power of distributed computing, federated learning can help develop a more accurate and efficient stroke assessment tool.

Federated learning allows models to be trained and enhanced across a multitude of decentralized devices or servers. This occurs without the need to transfer data to a central server, a departure from conventional machine learning, where data is gathered and transmitted to a central server or cloud for model training. This method proves especially valuable when data privacy is a priority or where a model’s limitations’ complexity and computational time are a factor, as it circumvents the necessity of centralizing sensitive or private data. Federated learning includes different approaches: horizontal federated learning, vertical federated learning, and federated transfer learning. These approaches offer flexible solutions for various scenarios. Horizontal federated learning is well-suited when there is significant overlap in user features between the two datasets but minimal user overlap. This means that the datasets may contain similar data types, but different individuals contribute to each dataset. By collaboratively training models on these datasets, while preserving data privacy, valuable insights can be gained from the collective knowledge without sharing raw data [27]. Vertical federated learning, on the other hand, is beneficial when the user features in the two datasets have minimal overlap, but there is significant user overlap. In this case, the datasets may contain different data types, but the individuals represented in the datasets are largely the same. By combining the information from both datasets in a privacy-preserving manner, a more comprehensive understanding of the shared user base can be obtained, enabling enhanced analysis and predictions [27]. In situations with limited users and user features overlapping between the two datasets, federated transfer learning comes into play. This approach employed transfer learning techniques to address the scarcity of data or tags in each dataset. Using pre-trained models on similar datasets, knowledge gained from one domain can be transferred and applied to improve learning and predictions in the target domain [27].

This paper proposes testing horizontal federated learning for the motion capture dataset. Since the different subjects or patients contribute to each node and the features of position and linear acceleration in the frequency domain, it is called horizontal federated learning. Each dataset is divided into different nodes and runs the federated learning techniques in each node/client independently from each other. The central server aggregates the model updates while preserving privacy and data security. Instead of sharing raw data, both centers exchange model updates with the central server during the federated learning process. These updates contain the insights and knowledge learned from each center’s training, specifically related to the accelerations and positional features. The federated learning approach allows the stroke rehabilitation center to gain valuable insights without directly exchanging raw data with the collaborating center. It ensures patient privacy, as the sensitive data remains within each center’s control. Federated learning can be applied to supervised and unsupervised machine learning models. Multiple devices or servers collaboratively learn, for instance, clustering models in federated clustering without sharing their raw data. Table 1 provides a concise overview of the key themes and findings from the literature review.

## 3. The Proposed PSA-FL-CDM Method

In post-stroke patient care, innovative methodologies are continually emerging to enhance the accuracy of assessments and treatment. This section introduces our centralized post-stroke severity assessment modified nonnegative matrix factorization model (PSA-MNMF) [23], a method that leverages consensus clustering and unsupervised machine learning techniques. Then, we propose the post-stroke assessment federated learning consensus driven model (PSA-FL-CDM), which incorporates federated learning and collaborative knowledge sharing among multiple centers to create a comprehensive global model for post-stroke assessment.

### 3.1. The Centralized Post-Stroke Severity Assessment Modified Nonnegative Matrix Factorization Model (PSA-MNMF)

The centralized post-stroke severity assessment modified nonnegative matrix factorization (PSA-MNMF) consensus clustering algorithm utilizes an input dataset and clustering algorithms to generate individual clustering methods. The input dataset undergoes baseline clustering algorithms, resulting in diverse outcomes due to each algorithm’s distinct assumptions, parameters, and randomness. A consensus matrix measures the similarity between the individual clustering methods. This matrix represents the agreement level between data points in the input data set. Applying NMF (nonnegative matrix factorization), the consensus matrix is factorized into two nonnegative matrices, *W* and *H*. This factorization is expressed as *C* ≈ *W* × *H*, where *C* represents the consensus matrix, *W* is the cluster assignment matrix, and *H* is the consensus centroid matrix. The rows of the consensus centroid matrix are then employed as input for a selected clustering algorithm, which differs from the individual baseline clustering algorithms. For this study, unsupervised machine learning was applied for each node/client. Eight clustering methods were employed, including the implemented and the PSA-MNMF consensus clustering methods introduced in our previous study [30]. This process yields a final consensus clustering solution. Ultimately, each data point is assigned to its corresponding consensus cluster.

**Figure 1:**
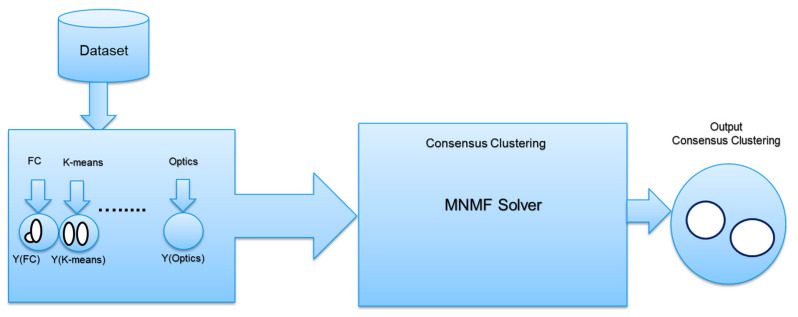
The PSA-MNMF model.

The post-stroke assessment-modified nonnegative matrix factorization (PSA-MNMF) was introduced in our previous work. PSA-MNMF is a novel consensus clustering algorithm designed to assess post-stroke severity levels using unsupervised learning and trunk displacement features in the frequency domain. By integrating data from wearable sensors (Xsens) and camera-based systems (Vicon), the study aims to provide a robust virtual assessment tool in the absence of labelled data and expert evaluation. Eight foundational clustering techniques are employed, including fuzzy c-means; k-means; self-organizing map (SOM); Gaussian mixture models; DBSCAN; and hierarchical, spectral, and OPTICS clustering. Consensus clustering merges these eight methods applied to the same dataset into a single, unified solution, capturing common patterns across different clusterings for a more stable and robust result. The efficacy of the proposed PSA-MNMF algorithm is demonstrated through improved accuracy, precision, recall, and F-scores across multiple datasets and clustering configurations. Notably, PSA-MNMF outperforms individual clustering methods and other consensus clustering algorithms like MCLA, showing particular strength in wearable sensor data due to lower noise levels [30]. Figure 1 illustrates the PSA-MNMF model. The dataset was input into multiple clustering methods. Then, a consensus clustering method (MNMF) was applied to derive the final output from the results of these eight different clustering methods.

### 3.2. The Post-Stroke Assessment Federated Learning Consensus Driven Model (PSA-FL-CDM)

**Figure 2:**
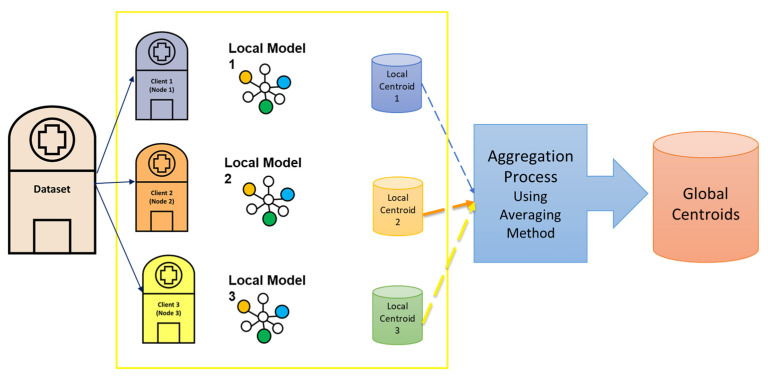
The Client-Server PSA-FL-CDM model.

**Figure 3:**
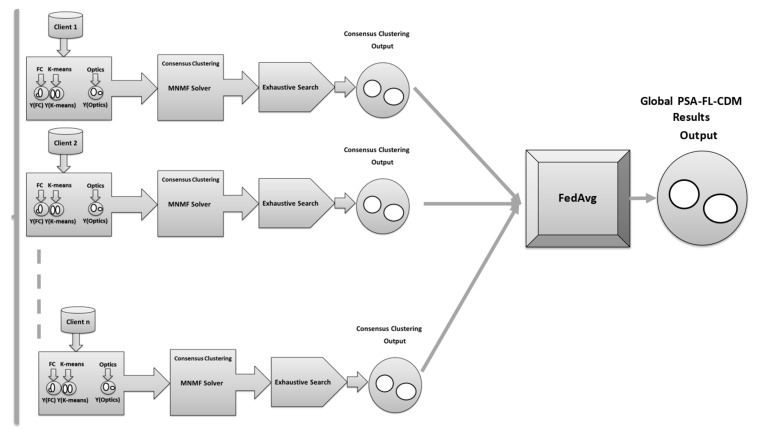
The Proposed PSA-FL-CDM.

This work proposes the post-stroke assessment federated learning consensus-driven model (PSA-FL-CDM). The cloud server receives the centers and corresponding labels in the PSA-FL-CDM algorithm. Once received, the cloud server applies the federated averaging (FedAvg) technique to construct the global model. The federated learning model employed in this study utilized parallelization techniques for client-server communication. In each client, the process of eight clusterings and the consensus clustering method PSA-MNMF for each node/client run in parallel. The local centers are then aggregated in the server, and the averaging method is applied to obtain the global centroids. The model scheme is presented in Figure 2. To elaborate further, the FedAvg technique aggregates the locally trained models from the various centers participating in the federated learning process. The cloud server averages the models’ parameters to create a global model that represents the collective knowledge of all the centers. Overall, the PSA-FL-CDM algorithm powers the FedAvg technique to enable collaboration and knowledge sharing among centers, creating a global model that benefits from the diverse contributions of the participating centers. Figure 3 illustrates the PSA-FL-CDM component of the proposed approach. Algorithm 1 provides an overview of the proposed algorithm. Algorithm 1 provides an overview of the proposed PSA-FL-CDM algorithm. Initially, the unlabelled dataset is divided among multiple clients or nodes. Each client runs the same clustering algorithm, such as k-means, with identical initialization parameters and a predefined number of clusters. The centers of these clusters are computed for each client. Subsequently, the PSA-MNMF model is applied to refine the cluster centers. Finally, the FedAvg method is utilized to finalize the centers of each cluster. The key steps of the algorithm are as follows:

1. Data Distribution: The unlabelled dataset is split and distributed across multiple clients or nodes.
2. Clustering Initialization: Each client initializes the clustering parameters identically, ensuring consistency across all nodes.
3. Clustering Computation: Each client performs clustering, computing the centers for their respective clusters.
4. PSA-MNMF Application: The PSA-MNMF model is applied to compute the refined centers of the clusters within each client.
5. Federated Learning Aggregation: The FedAvg method is used to aggregate the cluster centers from all clients, finalizing the centers for each cluster.
6. Final Labeling: The global centers are used to label the data points in the input dataset.
7. Performance Metrics: The algorithm concludes by returning performance metrics such as F-score, accuracy, precision, and recall.

**Algorithm 1:** PSA-FL-CDM**Input:** Unlabelled Dataset *A* = {*a*1, …, *an*}, randomly divide/distribute datasets to *M* client/node. Client datasets *M* = {*C*_1_, …, *C_M_*}*^M^_i_*_−0_.
Set of partitions *B* of each client *B* = {*b*1, *b*2, …, *bt*} and each partition *B* consists of a set of clustering *Dt* = {*d*_1_*^t^*, *d*_2_*^t^*, …, *d_k_^t^*} clustering methodology. Define the centers of each client as *X* = {*Xa*, *Xb*, *Xc*} (assuming number of cluster is 3 in the algorithm)
**Output:** The cloud server centers of the *K* cluster *W* = {*Wa*, *Wb*, *Wc*}
**Initialization:** Initialize the same parameters and hyperparameters for each Dt (each cluster with the same algorithm has the same initialization parameters to start with).
In each client or node compute the *X*-cluster = {The results of each clustering}
Describe the connectivity matric CM using the equation: $$CM_{ij}\left(b^{t}\right)=\{\begin{array}{c} 1\;\;\;\left(i,j\right)\in \;D_{k}\left(b^{t}\right)\\ 0\;\;\left(i,j\right)\notin \;D_{k}\left(b^{t}\right) \end{array}$$
Calculate *N_ixk_* that is a matrix with rows containing only one “1” and the remaining elements as “0”. Compute *NN^T^*, which is the result of checking whether the row index *i* belongs to the column index *k* in matrix *N*. Express *L* represents the matrix multiplication of *N* and its transpose.
**The Client Server**
/* The process of PSA-MNMF Consensus begins*/
**Step 1:** Compute the following PSA-MNMF optimization equation: ${\underset{N,\;\;L\geq 0}{\min}\|\tilde{CM}-\tilde{N}L{\tilde{N}}^{T}\|}^{2}$ where *N^T^N* = 1
**Step 2:** Update *N* At each iteration using this equation
$$N_{oi}\;\;\leftarrow \;N_{oi}\;\sqrt{\frac{{\left(CM\;N\;L\right)}_{oi}}{{\left(NN^{T}CM\;NL\right)}_{oi}}}\;$$
**Step 3:** Update *L* at each iteration, using $L_{ie}\;\;\leftarrow \;L_{ie}\;\sqrt{\frac{{\left(N^{T}CM\;N\right)}_{ie}}{{\left(N^{T}N\;L\;N^{T}N\right)}_{ie}}}$
**Step 4:** Repeat steps 1–4 for each node in parallel.
The cloud-server execution begins
/* The process of federated learning begins*/
**Step 5:** FedAvg begins using the following equation: $W_{k}^{t+1}\;\leftarrow \;\underset{k\in U_{t}}{\sum }\frac{X_{n}}{n}\;W_{k}^{t+1}$
**Step 6:** The final global centers of the PSA-FL-CDM solution is used to label each data point in the input dataset.
**Step 7:** Return performance metrics α (include F-score, accuracy, precision, recall).
**End**

## 4. Data, Materials, and Methods

This section provides a comprehensive overview of the post-stroke motion datasets used in this paper. The dataset utilized in this research is wearable sensors and camera-based sensors sourced from the U-limb datasets published in 2021. These open-source data were collected from various clinical settings following a standardized protocol [31].

A dataset derived from wearable-based sensors was utilized in a study at the University of Zurich (UZH) involving a subgroup of 20 stroke patients [32]. The data collected from these patients involved using 17 IMU sensor systems from the Xsens suite, which included a 3D angular magnetometer, 3D accelerometers, and a 3D gyroscope. The stroke patients’ functional movement assessment—upper extremity (FMA-UE) scores, with a mean of 46.00 ± 10.16, indicate that their impairments range from moderate to mild, as categorized in the study [33]. Both affected and non-affected hands were considered in the analysis. The participants in this study had an average age of 61.00 ± 10.69, consisting of 5 females and 15 males. Among them, 11 participants had affected right hands, 9 had affected left hands, and only one had a dominant left hand.

A dataset derived from camera-based sensors used in this study was collected by the Hannover Medical School (MHH) research group and involved healthy participants and stroke patients. The position data were captured using motion capture technologies, specifically a system consisting of 12 MX Vicon cameras (Vicon Motion System Ltd., Oxford, UK) operated by version 1.8.5 of the Nexus software. To track arm movements, 21 passive markers were attached to the upper body, including the thorax, upper arm, and forearm. For the stroke patient group, there were 20 participants, including 12 males and 6 females, with a mean age of 49.88 ± 16.92 years. The functional movement assessment—upper extremity (FMA-UE) score for this group was 17.75 ± 2.05, indicating a severe impairment level (category 3), as it fell below 29. Only the affected hand of each stroke patient was captured in this study. In addition to the stroke patients, there were 20 healthy participants, including 12 males, with a mean average age of 46.77 ± 15.25 years. The dominant hand was selected for testing in the healthy group, and 2 participants were left-handed. The decision to include the research group from Hannover Medical School was based on the same experiment and research protocol employed by the UZH group. Four specific grasping-action activities were selected for the current research, similar to our previous work in [30]. Each participant performed the four tasks three times, mirroring the sensor data collection. The dataset collected using the sensor-based data is referred to as dataset 1, while the dataset obtained through camera-based system measurements is labelled as dataset 2.

### 4.1. Data Preprocessing

**Figure 4:**
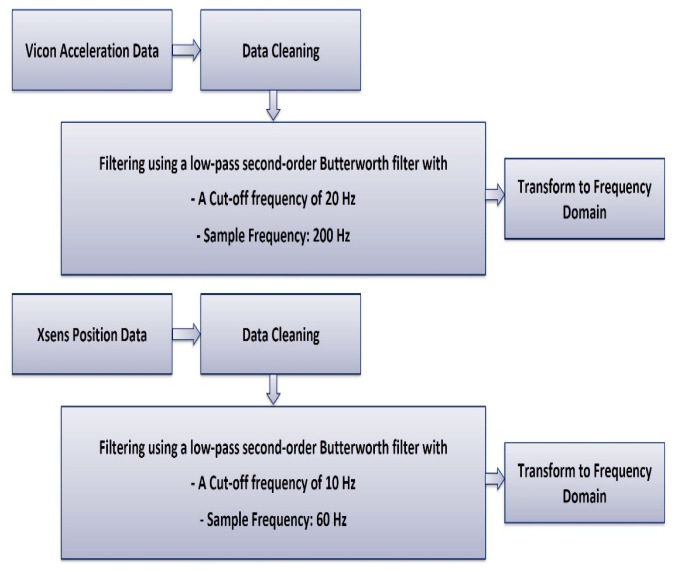
The preprocessing methodology utilized for position data.

The wearable sensor data were collected at a sampling frequency of 60 Hz. To ensure the accuracy of the data, a second-order Butterworth low-pass filter with a cutoff frequency of 10 Hz was applied. The camera-based position data was captured at a sample rate of 200 Hz and underwent filtering using a second-order Butterworth low-pass filter with a cutoff frequency of 20 Hz. This filtering was done to eliminate high-frequency noise components not generated by human movement. Additionally, we have included Figure 4, which outlines these steps employed for dataset 1 and dataset 2 to transform the position data into the frequency domain.

### 4.2. Wearable Sensors (Dataset 1)

For this research, we focused on selecting the 3D positions (x, y, z) of five major upper limb parts: hand, shoulder, upper arm, forearm, and sternum (T8). This choice resulted in 15 features comprising 3D predictor variables for each side of the body. The T8 data were utilized to measure trunk displacement. A relevant biomechanics equation was used to obtain the linear acceleration data from the position data.

### 4.3. Camera-Based Sensors (Dataset 2)

For the camera-based datasets, 3D positions were obtained from 9 markers at the wrist, ulnar, humerus, scapula, and trunk. These markers were selected for feature extraction, resulting in 27 features utilized for the camera dataset, each with three dimensions (x, y, z). To define trunk displacements, four markers positioned on the trunk were utilized. Similar to the approach employed in the wearable sensor dataset, acceleration was derived from the position data using a specific formula. In this study, the frequency domains of position and acceleration were subjected to testing and analysis, and this is a unique approach for both datasets. Examining data in the frequency domain offers the advantages of harnessing the entire dataset, ensuring that no data is overlooked, and elevating performance metrics. All adopted and consensus clustering models were run 100 times to reduce the variance. The exhaustive search method was applied to find the best combination of clustering methods.

### 4.4. Trunk Displacement Measurement

Measurements were conducted following the methodology described in a previous study [34], utilizing the T8 data from the wearable sensors and an average of four sensors positioned on the trunk from the camera-based sensors. Trunk displacements were determined based on variations in the position and orientation of the sensor at the sternum [35]. The mean value was computed in the initial step by averaging the first 10 data points. Subsequently, all the position data’s x, y, and z components beyond the initial 10 points were subtracted from this mean value. The following equation was then employed to calculate the desired value:

(1) $$Trunk\;Displacements=\sqrt{TD_{x}+TD_{y}+TD_{Z}}$$

This equation determined trunk displacement for each step based on the collected data. In this context, *TD_X_* represents the trunk displacement in the x direction (or front) at each step, while *TD_Y_* and *TD_Z_* represent the trunk displacements in the y and z directions, respectively. According to previous research, [36,37,38] trunk movements are compensatory movements observed in stroke patients during task performance. Cluster labelling was performed based on trunk displacement. For the camera-based dataset, four markers positioned on the trunk were chosen, and the displacement of each marker was computed using the aforementioned methodology. The average displacement of these four markers was then selected as the final trunk displacement for labelling each cluster.

### 4.5. Data Labeling

**Figure 5:**
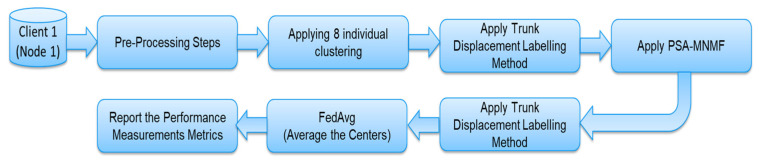
Client 1: The Proposed Procedure.

Our previous work [30] introduced a novel labelling approach to label clusters based on trunk displacement where higher displacement indicated a more severe stroke level. Stroke survivors often rely on trunk displacement to compensate for impaired motor function during daily activities [39,40]. The cluster with the lowest average displacement was selected to determine the healthiest or mildest level. The labelling results from each clustering technique were compared with each patient’s ground truth FMA (Fugl–Meyer Assessment) score. Both the centralized and federated learning approaches utilized the PSA-MNMF methodology. In the case of the federated learning model, the PSA-MNMF methodology was implemented individually in each node or client. Figure 5 Illustrates the proposed data labelling steps for one client.

**Figure 6:**
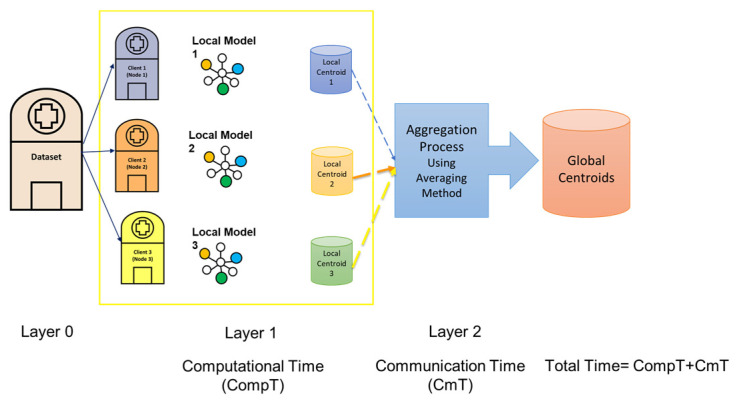
The total time calculation is demonstrated.

Figure 6 illustrates the recorded values for the overall computational time steps. The computational time (CompT) represents the maximum duration needed to run locally compared to each node. The communication time (CmT) corresponds to the time taken to perform the FedAVg process. The total time is calculated by adding CompT and CmT together. Next, we compare the total time required for the centralized model with the total time needed for the federated learning model, as explained.

## 5. Experimental Results

### 5.1. Performance Evaluation: Wearable Sensors (Dataset 1)

Table 2, Table 3, Table 4 and Table 5 present the outcomes of the proposed federated learning and centralized model of PSA_MNMF, focusing on dataset 1 with k = 2 and k = 3. Both methods are evaluated based on accuracy, precision, recall, and F-score. The evaluation of position data from dataset-1 is showcased in Table 2 and Table 4, highlighting the performance assessment. Furthermore, the acceleration data in the frequency domain is depicted in Table 3 and Table 5, emphasizing its representation. Table 2, Table 3, Table 4 and Table 5 reveal that the proposed federated learning model exhibited superior performance compared to the centralized PSA_MNMF methods, as assessed using two-level and three-level evaluations.

**Figure 7:**
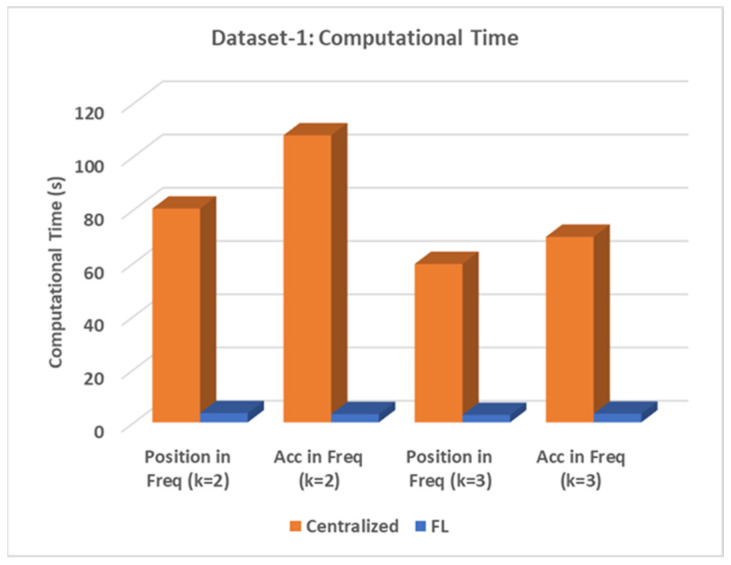
The total time comparison (dataset 1).

To compare the computational time between the proposed model and the centralized approach, Figure 7 and Table 6 are presented. It can be shown that the proposed model maintains high speed with comparable performance compared to the centralized approach.

### 5.2. Performance Evaluation: Camera-Based Data (Dataset 2)

Performance Evaluation with k = 2 and k = 3 is presented in Table 7, Table 8, Table 9 and Table 10. Both approaches undergo evaluation using metrics such as accuracy, precision, recall, and F-score. The performance evaluation of the position data in the frequency domain in dataset 2 is demonstrated in Table 7 and Table 9. These tables provide insights into the performance of the models. Table 8 and Table 10 illustrate the representation of acceleration data in the frequency domain. These tables showcase the characteristics of the acceleration data. 

**Figure 8:**
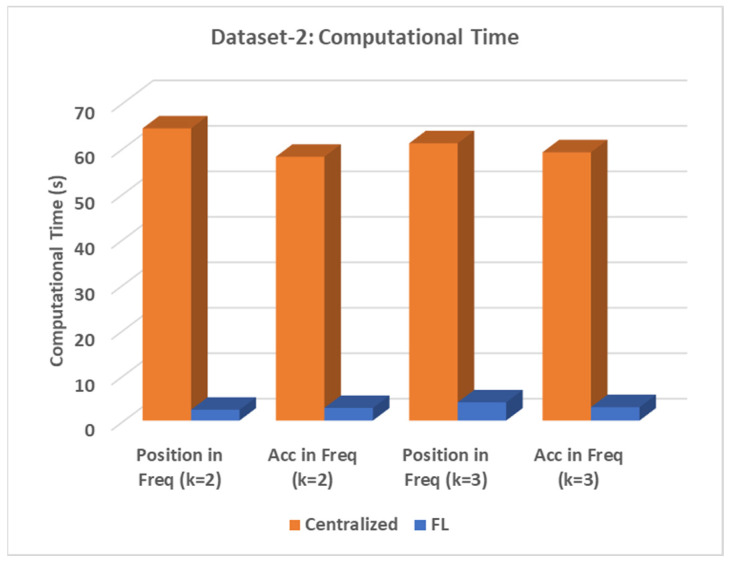
The total time comparison is demonstrated for dataset 2.

The results in Table 7, Table 8, Table 9 and Table 10 demonstrate that the proposed federated learning model outperformed the centralized PSA_MNMF methods in two- and three-level assessments. Figure 8 compares the computational time of the proposed and centralized models. This figure provides a visual comparison of the computational efficiency of the two approaches. Table 11 provides both the communication time and the computation time for dataset 2.

## 6. Discussion

The datasets of patients contain sensitive and confidential information, making privacy protection a top priority. However, to improve model performance, big data is necessary despite the significant challenges posed by the variation in patients’ datasets during training. Centralized data also incurs high computational costs, as all data need to be processed on a single server. Consequently, federated learning techniques have garnered attention from healthcare researchers, as they offer a solution that prioritizes privacy preservation by avoiding the need to share data with the server. In the previous study, the PSA_MNMF consensus clustering obtained the highest performance measured compared to other consensus clustering methods and individual clustering to assess the affected hand’s severity level. Therefore, federated learning was applied to automate stroke assessment for the first time, and unsupervised learning called PSA-MNMF consensus clustering was utilized.

In this study, we applied two levels of federated learning utilizing wearable and camera-based datasets. The PSA_MNMF consensus clustering is applied locally in each client or node, and then the center of each cluster is shared with the server and their labels where the aggregation model (FedAvg) occurs. The client-server federated learning strategy was applied for this study, and the type of this study was horizontal federated learning since the dataset was different. Still, the features were the same (position in the frequency domain and acceleration in the frequency domain). This study represents the pioneering exploration of a federated learning model in post-stroke assessment. We introduce the innovative concept of employing a consensus-driven model at each node within the artificial intelligence framework. Building upon our previous research, we leverage the benefits of utilizing data in the frequency domain and introduce a novel approach to trunk displacement labelling in post-stroke assessment. The results achieved through this approach have demonstrated promise. The performance evaluation outcomes for both dataset 1 and dataset 2 indicate that the federated learning (FL) model attains satisfactory performance compared to the centralized model. Moreover, the FL model demonstrates a significantly reduced computational time compared to the centralized model. These findings are encouraging as they highlight the FL model’s ability to safeguard patient privacy while improving model performance and reducing computational time compared to the centralized model. Integrating federated learning into automating stroke assessments in healthcare is an exciting and promising advancement. There is a strong recommendation to build upon the U-limb datasets published in 2021, where the data collection protocol has been used to collect data from stroke patients worldwide. Expanding the implementation of this protocol to different rehabilitation centers worldwide while safeguarding patients’ privacy can significantly improve the performance of the models. This collaborative approach holds immense potential for driving forward stroke research globally, benefiting patients worldwide.

Our study exhibited a notable strength by utilizing data from stroke patients. Additionally, the implementation of consensus clustering methods contributed to reducing dataset heterogeneity, minimizing bias, and establishing a more resilient and consistent model. This approach also effectively decreased communication costs. Specifically, employing the PSA-MNMF consensus clustering method resulted in faster convergence of data, thereby enhancing the efficiency of the process. In addition, our study stands out as the pioneering endeavour in implementing unsupervised learning techniques and applying federated learning, specifically in stroke assessment. We extended our analysis to include camera-based and wearable sensor datasets, comprehensively examining different data sources. However, a significant limitation of our study stems from the scarcity of available datasets. Since automating stroke assessment is still in the research phase, there is a shortage of data collected using similar protocols compared to the medical images area. This constraint hampers the comprehensive analysis and limits the generalizability of our findings. Finally, evaluating and testing different network conditions should be considered as future work for this study.

Exploring the application of unsupervised learning techniques, particularly consensus clustering, on real-time motion and position data collected from stroke patients presents a novel and uncharted endeavor, considering the difficulty in finding similar protocol-based datasets. Moreover, it is important to note that automated stroke assessment is yet to be implemented in clinical environments. Although this study did not include the implementation across multiple rehabilitation centers due to the aforementioned challenges, the concepts were tested on two distinct types of datasets. The application of FL in healthcare, and hopefully in rehabilitation, holds great potential for advancing automatic prediction, diagnosis, and assessment systems. These advancements aim to facilitate the development of robust, scalable, and privacy-preserving health services. Notably, distributed learning is expected to foster larger-scale and collaborative healthcare and rehabilitation systems, potentially enabling fully decentralized diagnosis operations instead of relying on centralized analytics in data centers.

## 7. Conclusions and Future Directions

Federated learning offers several advantages over traditional centralized consensus clustering methods. It enables clustering models to be trained on data dispersed across multiple devices or servers, including sensitive data such as healthcare or financial information, without compromising data privacy or security. Moreover, federated learning can help reduce the costs associated with communication and computation, as only the updated model parameters are sent across the network instead of the entire dataset, as required in traditional centralized consensus clustering approaches. For future work, exploring alternative aggregation methods compared to the commonly used FedAvg, which was employed to aggregate the centers in this study, would be valuable [36]. Additionally, conducting a comparative analysis was impossible since no unsupervised learning technique was utilized in the stroke field and the context of federated learning. Therefore, applying different aggregation methods and comparing the results with those obtained in this research is highly recommended. Given the privacy-preserving nature of federated learning, employing this approach in stroke or rehabilitation settings is remarkably advisable to develop more refined and accurate models. In healthcare, precision and accuracy hold significant importance, and implementing big data is highly recommended, especially due to the diversity of patients’ data. Such efforts would contribute to advancing the field and refining the understanding of federated learning’s potential in stroke assessment, rehabilitation, and beyond [39,40].

## Figures and Tables

**Table 1:** A Summarized Literature Review.

Ref	FL Strategy/Category	ML	Dataset	Strength	Limitation	Naming	Performance Evaluation
Ju et al. (2020) [26]	Client-server computing, FedAvg/ Horizontal FL	NN, RF, LG, XGBoost	EHR	Predict the risk of stroke. Sharing data from different hospitals in the city	No other performance metrics such as accuracy, F-measure reported	Fed-AI Stroke Prediction	AUC
Wu et al. (2022) [19]	Client-server computing (Cloud-edge based), FedAvg/Vertical FL	Deep learning	Collected from a smartphone—Healthy Subject	Emphasis is placed on imbalanced and highly skewed data distribution.	Healthy Subjects aged between 20 and 47 years old have been used.	FedHome GCAE	Accuracy
Chen et al. (2010) [21]	Client-Server computing, FedAvg/ Federated Transfer Learning	CNN	Wearable dataset for healthcare—Activity Recognition UCI smartphone dataset	For HAR, using federated transfer learning	This study does not verify or analyze communication and training costs. No patient data	FedHealth	Accuracy Mean F1 (Micro F1 score)
Liu et al. (2019) [18]	Cloud Server Client-Server computing/ Horizontal FL	NLP	Clinical notes of obesity and comorbidities phenotyping from different hospitals	The pioneering application of FL in clinical NLP.	Privacy preservation for the federated model is not considered in the study.	Two-stage Federated Obesity and Phenotyping Analytics	Precision, Recall, F1-Score
Yan et al. (2021) [13]	Client-server computing, FedAvg/ Vertical FL	Deep learning	Medical images dataset (from prostate images)	Addressing cross-client variation by image transformation onto a common space	The study does not investigate learning accuracy.	A variation-aware federated learning (VAFL) framework	Accuracy, AUC
Silva et al. (2019) [15]	Data-center Client-server computing/Horizontal FL	PCA	MRI Images ADNI, PPMI, MIRIAD and UK Biobank	Multiple databases from various centers were utilized	No practical aspects of federated MRI training.	Alternating Direction Method of Multipliers (ADMM)	MSE
Kumar et al. (2021) [16]	Data-center Client-server computing/Horizontal FL	Deep Learning	CT scan of COVID patients from different hospitals	Deal with heterogeneity of data by utilizing normalization and deployed capsule-network-based segmentation.	No detailed analysis was described.	Blockchain-Federated-Learning and Deep Learning Models	Recall/sensitivity and Accuracy
Yan et al. (2021) [13]	Data-Center Client-server computing/Horizontal FL	Deep Learning	Chest X-ray Images	Comparison was made between their proposed model and MobileNet_v2, ResNet18, and ResNeXt.	The convergence of FL has not been performed.	Covid FL model Covidnet	Accuracy
Zhang et al. (2021) [14]	Client-server computing/Horizontal FL	Deep Learning	Medical images X-rays and CT scans	It was addressed by minimizing the communication cost associated with model updates and transfers.	The study does not report on learning efficiency metrics such as latency.	Dynamic Fusion-based federated learning	Accuracy
Xu et al. (2022) [28]	Client-Server Computing FedAvg/Horizontal FL	Deep learning	Mobile Health Data	Used six hospital datasets. Three models were compared (DNN, DFM, DMVM).	The participant’s performance was not considered.	DeepMood	Accuracy
Tedeschini et al. (2022) [17]	Fully-decentralized FL (Consensus-driven FL) Serverless and peer-to-peer communication/Transfer Learning FL	Deep Learning	Medical images for brain tumor segmentation	Utilize the FL to process different countries communicating via the internet. Report the accuracy vs. latency trade-offs.	There is no reporting of any other performance matrix, such as accuracy, F1-Score, etc.	Message Queuing Telemetry Transport (MQTT)	DSC
Arikumar et al. (2022) [29]	Edge-Server Computing/Horizontal FL	DRL, BiLSTM	Wearable data (Smart Watch)	Accuracy, computational costs, memory usage, and transmission time were reported. Used BiLSTM	Labelling is done with whole datasets. Security treatment did not consider	Federated Learning-Based Person Movement Identification	Accuracy, Transmission time

**Table 2:** Comparison of Accuracy, Precision, Recall, and F-score for the Proposed Federated Learning and Centralized Models on Dataset 1 (Position in the Frequency Domain with k = 2).

k = 2, Number of Node = 15	Centralized	FL
Accuracy	71.46%	65.26%
P	69.14%	67.77%
Recall	71.46%	65.26%
F-Score	68.66%	66.14%
Total Computational Time (s)	80.3	3.47

**Table 3:** Comparison of Accuracy, Precision, Recall, and F-score for the Proposed Federated Learning and Centralized Models on Dataset 1 (Acceleration in the Frequency Domain with k = 2).

k = 2, Number of Node = 15	Centralized	FL
Accuracy	61.72%	66.59%
P	63.44%	74.82%
Recall	61.72%	66.59%
F-Score	62.43%	67.79%
Total Computational Time (s)	107.85	3.11

**Table 4:** Comparison of Accuracy, Precision, Recall, and F-score for the Proposed Federated Learning and Centralized Model on Dataset 1 (Position in the Frequency Domain with k = 3).

k = 3, Number of Node = 20	Centralized	FL
Accuracy	54.2%	55.53%
P	59.81%	62.21%
Recall	54.2%	55.53%
F-Score	55.88%	57.39%
Total Computational Time (s)	59.51	2.79

**Table 5:** Comparison of Accuracy, Precision, Recall, and F-score for the Proposed Federated Learning and Centralized Model on Dataset 1 (Acceleration in the Frequency Domain with k = 3).

k = 3, Number of Node = 11	Centralized	FL
Accuracy	53.09%	55.53%
P	54.48%	63.81%
Recall	53.09%	55.53%
F-Score	53.11%	57.39%
Total Computational Time (s)	69.7	3.22

**Table 6:** Communication and Computation Time for Dataset 1.

Wearable Sensor Datasets				
Time Record	Position k = 2	Acceleration k = 2	Position k = 3	Acceleration k = 3
Computation Time	3.47	3.11	2.46	2.92
Communication Time	0.3	0.35	0.33	0.3
Total Time	3.77	3.46	2.79	3.22

**Table 7:** Comparison of Accuracy, Precision, Recall, and F-score for the Proposed Federated Learning and Centralized Models on Dataset 2 (Position in the Frequency Domain with k = 2).

k = 2, Number of Node = 25	Centralized	FL
Accuracy	56.87%	69.93%
P	56.99%	70.92%
Recall	56.87%	69.93%
F-Score	56.67%	69.55%
Total Computational Time (s)	64.25	2.39

**Table 8:** Comparison of Accuracy, Precision, Recall, and F-score for the Proposed Federated Learning and Centralized Models on Dataset 2 (Acceleration in the Frequency Domain with k = 2).

k = 2, Number of Node = 20	Centralized	FL
Accuracy	68.38%	74.93%
P	68.38%	74.98%
Recall	68.38%	74.93%
F-Score	67.37%	74.92%
Total Computational Time (s)	58.02	2.79

**Table 9:** Comparison of Accuracy, Precision, Recall, and F-score for the Proposed Federated Learning and Centralized Models on Dataset 2 (Position in the Frequency Domain with k = 3).

k = 3, Number of Node = 10	Centralized	FL
Accuracy	30.06%	40.09%
P	30.49%	42.63%
Recall	30.06%	40.09%
F-Score	30.24%	41.05%
Total Computational Time (s)	60.97	4.01

**Table 10:** Comparison of Accuracy, Precision, Recall, and F-score for the Proposed Federated Learning and Centralized Models on Dataset 2 (Acceleration in the Frequency Domain with k = 3).

k = 3, Number of Node = 10	Centralized	FL
Accuracy	34.73%	42.18%
P	37.89%	52.56%
Recall	34.73%	42.18%
F-Score	35.59%	44.56%
Total Computational Time (s)	58.99	2.95

**Table 11:** Communication and Computation Time for Dataset 2.

Camera Datasets				
Time Record	Position k = 2	Acceleration k = 2	Position k = 3	Acceleration k = 3
Time Record	2.05	2.43	4.01	2.95
Computation Time	0.34	0.36	0.5	0.5
Communication Time	2.39	2.79	4.51	3.45

## Data Availability

The original data presented in the study are openly available at https://doi.org/10.1093/gigascience/giab043, accessed on 29 July 2024 and https://doi.org/10.5281/zenodo.3713449, accessed on 29 July 2024.

## Abbreviations

FL	Federated Learning
ML	Machine Learning
FMA	Fugl-Meyer Assessment
MRI	Magnetic Resonance Imaging
PSA-MNMF	Post Stroke Assessment-Modified nonnegative Matrix Factorization
IOT	Internet of Things
EEG	electroencephalography
AI	Artificial Intelligence
IMUs	Inertial Measurements Units
MQTT	Message Queuing Telemetry Transport
ANN	Artificial Neural Network
AUC	Area Under the Curve
MSE	Mean-Squared Error
DSC	Dice Similarity Coefficient
RF	Random Forest
LR	Logistic Regression
XGBoost	eXtreme Gradient Boosting
NN	Neural Networks
HER	Electronic Health Record
HAR	Human Activity Recognition
NLP	Natural Language Processing
PCA	Principal Component Analysis
DRL	Deep Reinforcement Learning
BiLSTM	Bidirectional Long Short-term Memory
DSC	Dice Similarity Coefficient
